# Supplementary material for: Imaging-Guided Delivery of a Hydrophilic Drug to Eukaryotic Cells Based on Its Hydrophobic Ion Pairing with Poly(hexamethylene guanidine) in a Maleated Chitosan Carrier
Source: Molecules. 2021 Dec 7;26(24):7426. doi: 10.3390/molecules26247426 (PMC8703758; doi:10.3390/molecules26247426)
Supplement: Supplementary file 1 [file molecules-26-07426-s001.zip › molecules-1464599-supplementary.pdf]

Supplementary Information to paper

Imaging-Guided Delivery of a Hydrophilic Drug to Eukaryotic Cells Based on its Hydrophobic Ion Pairing with Poly(hexamethylene Guanidine) in a Maleated Chitosan Carrier

Sofia A.Zakharenkova, Marina I.Lebedeva, Alexandra N.Lebedeva, Irina A.Doroshenko, Ksenya Yu.Vlasova, Anastasiya A.Bartoshevich, Vladimir M. Senyavin, Sergey S.Abramchuk, George G.Krivtsov, Alexander A.Ezhov, Tatyana A.Podrugina, Natalya L.Klyachko, Mikhail K.Beklemishev

Synthesis of the dye [1]

One gram (4.78 mM) of 1,1,2-trimetyl-1H-benzo[e]indole and 1.27 g (4.78 mM) of 11-bromoundecanoic acid were heated at 100 °C for 12 h. After cooling to room temperature, an excess of diethyl ether was added and the precipitate was filtered off. 2.32 g of dark green powder was obtained (yield 92%); melting point 195 °C–200 °C. NMR 1H ($CDCl_3$, δ, ppm, J/Hz): 1.23 (br.s., 8H), 1.35 (br.s., 2H), 1.47 (br.s., 2H), 1.52–1.58 (m, 2H), 1.85 (s, 6H), 1.97 (br.s., 2H), 2.28 (t, 2H, $^3J_{HH}$=7.3), 3.18 (s, 3H), 4.81 (br.s., 2H), 7.61–7.82 (m, 3H), 8.07 (m, 3H). One equivalent of the received powder and 1.2 equivalents of N-((1E,3Z)-3-(phenylimino)prop-1-en-1-yl) aniline hydrochloride were dissolved in 1.5 mL of acetic anhydride and the reaction mixture was heated at 120°C for 30 min. Then 1.7 equivalents of ammonium salt were dissolved in 1.5 mL of pyridine, added to the reaction mixture and heated for 30 min. After cooling to room temperature, an excess of diethyl ether was added and the precipitate was filtered off. The target compound was purified by column chromatography on silica gel (eluent: CH_2Cl_2/MeOH, 30:1 v/v). The carbocyanine dye was obtained as a dark blue powder.

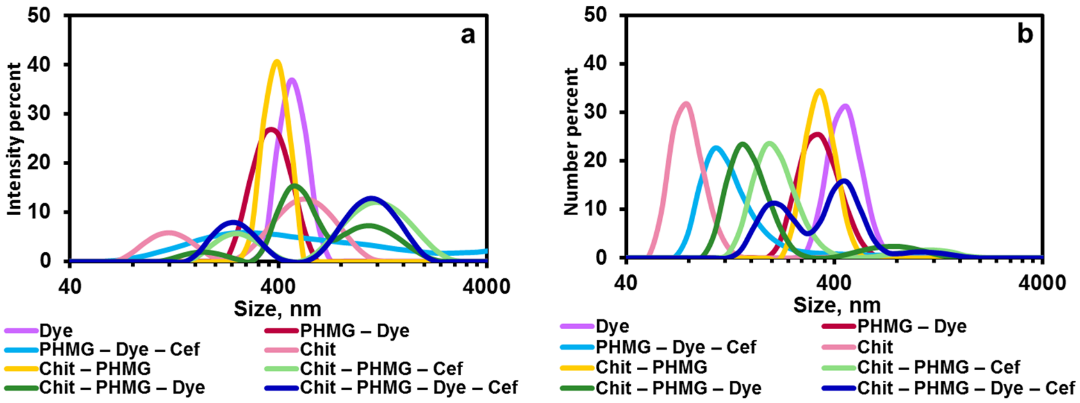

Figure S1. Sizes of container particles *chitosan – PHMG – dye – ceftriaxone* and their components obtained by dynamic light scattering technique: *a* – by intensity, *b* – by number. **Chit** – maleated chitosan, **PHMG** – poly(hexamethylene guanidine), **Cef** – ceftriaxone. All chitosan-containing systems were cross-linked by glutaraldehyde (1 day at room temperature). Concentrations of all individual components are the same as in the protocol for obtaining the cross-linked containers.

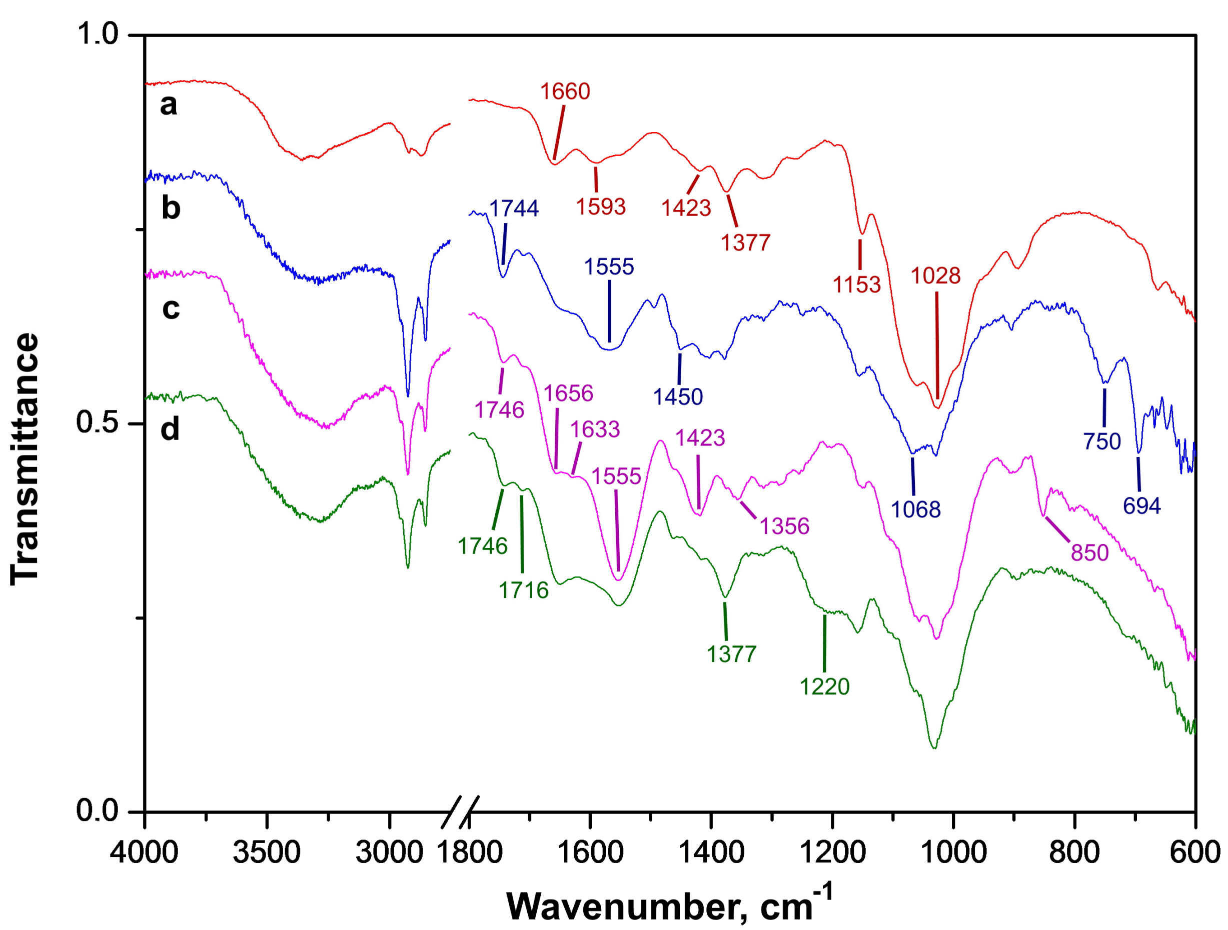

Figure S2. FT-IR spectra of the solid samples of chitosan (*a*), carboxymethylated (*b*), maleated (*c*), and maleated sulfated (*d*) chitosan.

Upon carboxymethylation (curve *b*), the bands of methylene group stretching and bending vibrations appear in the spectrum at 2925, 2853, 1450 and 750 cm^{-1}, as well as those of carboxyl group at 1744, 1068 and 694 cm^{-1}. The δ(NH) band at 1555 cm^{-1} remarkably rises suggesting a partial functionalization of amino-group. Maleation of chitosan sample (curve *c*) leads to further enhancement of this band intensity and to the red shift of ν(OH) band due to harder hydrogen bonding in network. A band appears at 850 cm^{-1} which can be assigned to out-of-plane δ(OH) or δ(=CH) vibration. Sulfo-group addition manifests itself in appearing of bands at 1377 and near 1220 cm^{-1} (curve *d*). The peak at 1746 cm^{-1} in all modified chitosans can be attributed to the ν(C=O) vibraton of carboxylic group. Another weak ν(C=O) band at 1716 cm^{-1} can correspond to the maleate ester due to the reaction of the anhydride by oxygen rather than nitrogen atom [2].

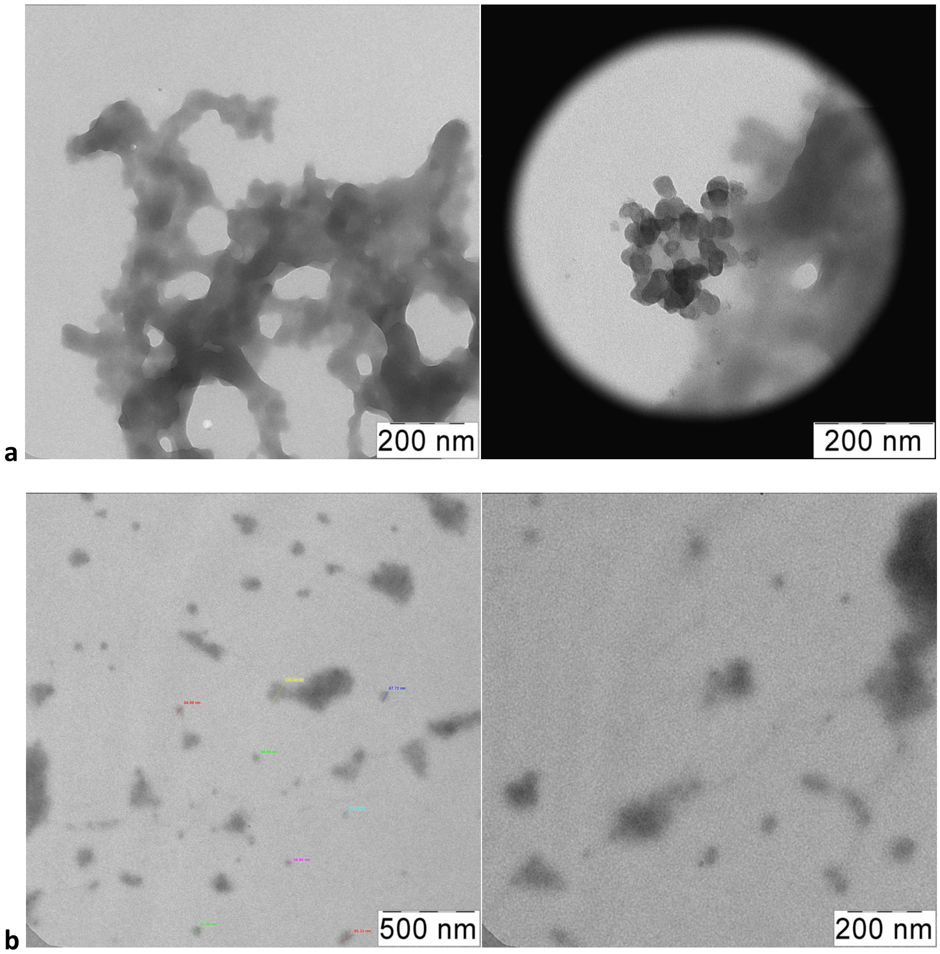

Figure S3. Transmission electron microscope images of containers *maleated chitosan – PHMG – dye* (a) and **non-cross-linked** aggregates *PHMG – ceftriaxone – dye – maleated chitosan* (b). The solutions were evaporated to dryness on a TEM copper grid.

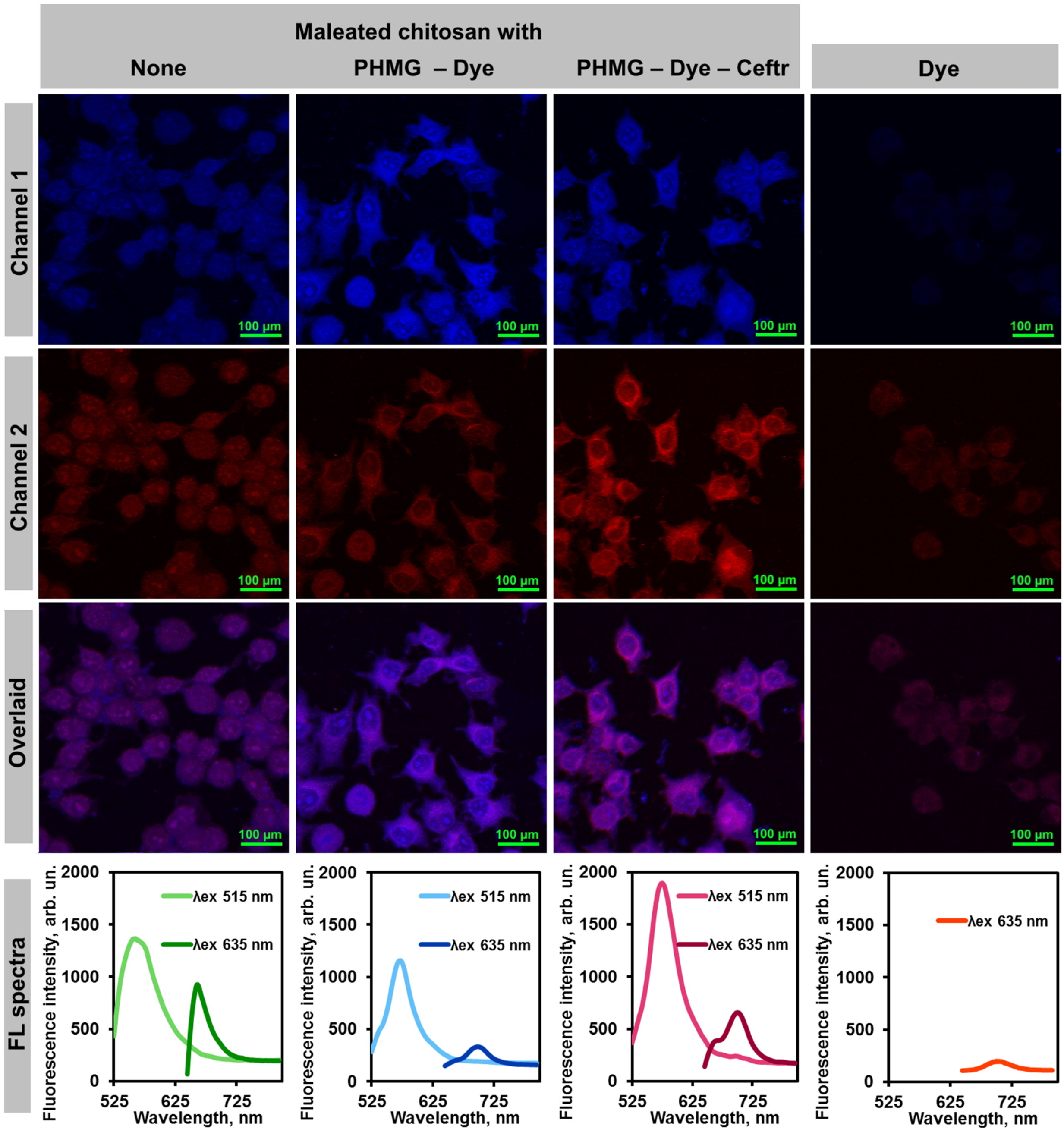

Figure S4. CLSM images of the cells after contact with the suspension of containers *ceftriaxone – PHMG – dye – maleated chitosan* (3rd column from the left) or the respected control solutions (maleated chitosan alone*, PHMG – dye – chitosan,* and dye alone) and the fluorescense spectra obtained in CLSM.

All chitosan-containing solutions were cross-linked with glutaraldehyde. Emission in channel 1 (ex 515 nm, registration at 540–590 nm) refers to Rhodamine-labeled chitosan. Emission in channel 2 (ex 635 nm, registration at 655–675 nm) for PHMG alone is explained by the cells autofluorescence [3]; maximum of its emission intensity is at 670 nm. Weak emission in channel 2 in the images with the dye-PHMG is explained by dye aggregation in water, where it is weakly fluorescent; the dye emission maximum is at 700 nm. Emission in channel 2 for the *ceftriaxone – PHMG – dye – maleated chitosan* system (3rd column) corresponds to the dye solubilized in the aggregate *PHMG – ceftriaxone*, its emission maximum is at 700 mn, and a weak band at 670 nm corresponding to the cells autofluorescence is also visible.

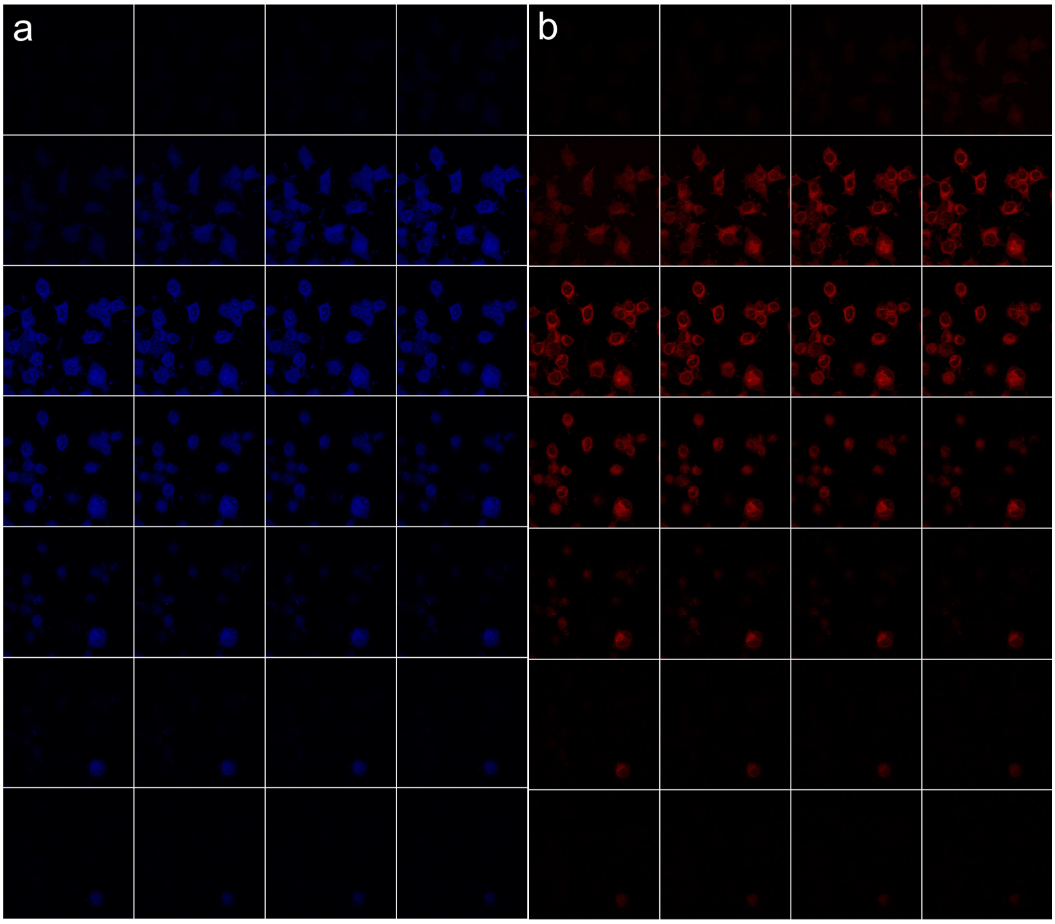

Figure S5. All optical sections of CLSM Z-stack images of cells incubated with containers *PHMG – dye – ceftriaxone* in maleated chitosan. Fluorescence was excited at (*a*) 515 nm and measured in channel 1; (*b*) 635 nm and measured in channel 2. Photo field size: 640×640 µm.

References

1. Zakharenkova, S.A.; Katkova, E.A.; Doroshenko, I.A.; Kriveleva, A.S.; Lebedeva, A.N.; Vidinchuk, T.A.; Shik, A.V.; Abramchuk, S.S.; Podrugina, T.A.; Beklemishev, M.K. Aggregation-based fluorescence amplification strategy: "turn-on" sensing of aminoglycosides using near-IR carbocyanine dyes and pre-micellar surfactants. *Spectr. Acta A*, **2021**, *247*, 119109. https://doi.org/10.1016/j.saa.2020.119109.

2. Septiawan, M.R.; Permana, D.; Sabarwati, S.H.; Ahmad, L.O.; Ramadhan, L.O.A.N. Functionalization of Chitosan with Maleic Anhydride for Proton Exchange Membrane. *Indones. J. Chem*. **2018**, *18*, 313-320 https://doi.org/10.22146/ijc.33141.

3. Lifante, J.; Shen, Y.; Ximendes, E.; Rodríguez, E.M.; Ortgies, D.H. The role of tissue fluorescence in *in vivo* optical bioimaging. *J. Appl. Phys.* **2020**, *128*, 171101. https://doi.org/10.1063/5.0021854.
